# Suffering Job Insecurity: Will the Employees Take the Proactive Behavior or Not?

**DOI:** 10.3389/fpsyg.2021.731162

**Published:** 2021-09-21

**Authors:** Xun Yao, Meng Li, Huiqin Zhang

**Affiliations:** ^1^Business School, Southwest Minzu University, Chengdu, China; ^2^College of Management Science, Chengdu University of Technology, Chengdu, China

**Keywords:** job insecurity, proactive behavior, self-efficacy, cognitive appraisal, social cognitive theory, cognitive appraisal theory

## Abstract

Job insecurity is considered an important antecedent of an employee’s creativity. Though, the relationship between job insecurity and proactive behavior has been neglected in previous human resources management studies. The aim of this study is to explore the influence of job insecurity on employees’ proactive behavior and its mechanism. Based on the social cognitive theory and cognitive appraisal theory, two types of cognitive appraisal of employee’s job insecurity (hindrance vs. challenge) as mediator variables of job insecurity and proactive behavior association. In addition, the moderator roles of self-efficacy are examined. This study is carried out with 257 employees from Chinese firms to examine the hypothesized moderated mediation model by using the hierarchical regression analysis and the bootstrap. The results showed a different effect of job insecurity depending on its conceptualization. The results show that job insecurity has a negative effect on employees’ proactive behavior. At the same time, cognitive appraisal of employees’ job insecurity mediated the association between job insecurity and employee’s proactive behavior. Self-efficacy not only moderates the relationship between job insecurity and cognitive appraisal but also moderate the cognitive appraisal’s mediation effect between job insecurity and proactive behavior. The study’s theoretical and practical contributions and future research are discussed.

## Introduction

At present, the fierce market competition and the adjustment of industrial structure make the organization face the tremendous reform pressure of adjusting organizational structure to adapt to the current market environment. At the same time, due to the great impact of the COVID-19 epidemic in 2020, organizational consolidation and restructuring, layoffs, temporary and short-term employment are becoming more and more significant, leading to employees’ concerns about the uncertainty of their future jobs (Hirsch and De Soucey, 2006). In other words, employees’ job insecurity has been widespread in all kinds of organizations (Saif et al., 2020).

According to the above content, scholars have conducted a mufti-perspective discussion on job insecurity. Early studies mostly focused on the internal physical and mental effects of job insecurity on employees’ psychological stress, organizational commitment (Chirumbolo et al., 2017), turnover intention (Staufenbiel and Konig, 2010; Chirumbolo et al., 2017), well-being (De Witte et al., 2016; Sora et al., 2018), job satisfaction (Callea et al., 2016; Chirumbolo et al., 2017), burnout (Lastad et al., 2018; Saif et al., 2020), and physical health (De Witte et al., 2016; Lastad et al., 2018). With the study of the internal effects of the employee, the research on the outcome variables of job insecurity has gradually extended to the field of behavior. Scholars have studied the influence of job insecurity on employees’ actual turnover behavior (Richter et al., 2020), workplace deviance (Xiao et al., 2018), counterproductive behavior (Nawrocka et al., 2021), and organizational citizenship behavior (Lam et al., 2015) from different perspectives. The studies on the influence of job insecurity on employees’ innovative behavior (Teng et al., 2019) have also sprung up as organizations attach increasing importance to “innovation.” There are no researchers that have been found to pay attention to the impact of job insecurity on proactive behavior. Therefore, this research aims to fill this research gap and explore the relationship between job insecurity and employees’ proactive behavior and its mechanism.

As we all know, human behavior is guided by motivation. At the same time, it will be affected by the environment and personal characteristics of the employees. According to Lewin’s field theory, a person’s behavior depends on the interaction between the individual and his environment (Lewin, 1951). Work stressors (such as time pressure, situational pressure, etc.) in the organizational environment will affect the proactive behavior of employees. Therefore, as an important source of chronic work stress in an organization (Richter et al., 2014), job insecurity will inevitably have an impact on the proactive behavior of employees.

However, it is not difficult to find that employee’s attitudes and reactions to job insecurity caused by various reasons show a polarized phenomenon. Some employees will be diligent and cheerful, break their work boundaries, try their best to improve work performance, and show proactive behavior to cope with their job insecurity. The others will be lazy, “broken cans,” and more passive. Most of the existing studies on job insecurity focus on the negative effects of job insecurity. A large number of studies have shown that job insecurity will have a variety of negative effects on employees, such as: reduce the level of organizational commitment of employees (Chirumbolo et al., 2017), increase their turnover intention (Staufenbiel and Konig, 2010; Chirumbolo et al., 2017), reduce their job satisfaction (Callea et al., 2016; Chirumbolo et al., 2017) and well-being (De Witte et al., 2016; Sora et al., 2018), and damage their physical and mental health (Chirumbolo et al., 2017; Lastad et al., 2018). It also negatively impacts employees’ family and social sphere (Barling et al., 1998; Jiang et al., 2019). Therefore, Sverke et al. (2002) believe that enterprises should try to avoid employees’ job insecurity to reduce the negative effects of job insecurity. However, the positive influence of job insecurity is funded by scholars. For instance, Teng and his colleagues found that job insecurity can also improve employees’ creativity and promote innovative behaviors (Teng et al., 2019).

The social cognitive theory systematically explains the generation process of individual behavior from an individual cognition perspective (Bandura, 2001), which points out that human activities are interacted by the external environment, individual cognition and other individual characteristics, and individual behavior. Precisely, individuals form expectations and action plans based on their cognitive appraisal of themselves and the environment, and through self-management to display corresponding behaviors to achieve the purpose of influencing the environment. We can speculate that employees’ cognitive appraisal of job insecurity will inevitably impact proactive behavior. However, whether this effect is positive or negative remains to be further discussed. Otherwise, Ohly and Fritz (2010) proved that chronic job stressors such as job characteristics would have an impact on employees’ proactive behavior via challenge appraisal. Therefore, we selected employees’ cognitive appraisal of job insecurity as a mediating variable. At the same time, some researchers indicated that self-efficacy influences individual behavior through individual cognition, emotion, and the decision-making process (Bandura, 2012). That is to say, self-efficacy will impact employees’ proactive behavior through employees’ cognitive appraisal. Therefore, in this study, self-efficacy as an individual characteristic variable was comprehensively considered to explore its moderating effect on the entire influencing mechanism.

To sum up, this study constructs a moderated mediation model, which takes proactive behavior as a result variable, based on social cognitive theory, and introduces employees’ self-efficacy and cognitive appraisal (including hindrance appraisal and challenge appraisal) as moderating and mediating variables. We used the questionnaire survey to collect the data, test the hypothesis by using the hierarchical regression analysis. It aims to explore the effect and mechanism of job insecurity on proactive behavior, open the “black box” of the interaction mechanism between job insecurity and proactive behavior, further enrich the theoretical achievements in related research fields. At the same time, put forward targeted management enlightenment for enterprises to stimulate employees’ proactive behavior to cope with the torrent of market competition.

## Theory and Hypotheses

### Job Insecurity and Proactive Behavior

Greenhalgh and Rosenblatt (1984) studied job insecurity for the first time. They believed that job insecurity is the concern of employees about the possible loss of continuity of a particular job in the future, including the permanent loss of the job itself and the loss of some positive or essential characteristics of the job. The concept of job insecurity is future-oriented and reflects a prediction of the future. Therefore, the job insecurity studied in this paper is not the actual loss of job or job characteristics, but the attitude and reaction of employees to the possible “loss of job or job characteristics” in the future (Boswell et al., 2014). This kind of job insecurity often leads to different degrees of psychological stress (Naswall et al., 2005) and is one of the most prominent social psychological risks in the workplace (Jiang and Lavaysse, 2018).

At present, there are many kinds of research on proactive behavior, but there is no consensus on its conceptual connotation. Proactive behavior refers to the spontaneous and prospective behavior in which employees take positive actions to change or improve themselves and their situation (Parker et al., 2006). The definition of proactive behavior is not limited to in-role behavior, but also includes out-of-role behavior, such as responsible behavior, voice behavior, job shaping, and construct social networks, etc (Parker and Collins, 2010). The most important characteristic that distinguishes proactive behavior from other behaviors is its characteristics of spontaneity, foresight, and transformation. Therefore, the main research on proactive behavior is why individuals want to take the initiative to improve themselves and the environment, improve and impact individuals and organizations, and so on.

This study discusses the “cause-driven” mechanism of job insecurity on proactive behavior starting from the driving mechanism of proactive behavior. According to the planned behavior theory, behavioral intention is the best way to predict and explain individual behavior, which can directly affect individual behavior (Ajzen, 1991). The behavioral intention here is consistent with the individual motivation, which will lead the individual to take the corresponding behavior to achieve the ultimate goal. When employees perceive that their future jobs or specific job characteristics are “threatened,” some employees believe that they can reduce the possibility of losing their jobs or job characteristics in the future by actively improving themselves and the situation. To reduce job insecurity, employees may try their best to show proactive behaviors to improve work performance, increase their weight in the superior leadership and organization, reflect their value, and reduce such “threats” (Lam et al., 2015). That is to say, job insecurity may motivate employees to exert their subjective initiative and promote their proactive behavior. However, when some employees faced with the pressure that they may lose their jobs or specific work characteristics in the future, on the one hand, they are worried about more adverse effects on them due to more mistakes in the process of doing things on their initiative; on the other hand, they are afraid that they may be ostracized or even retaliated against for “doing too much” which threatens the interests of others (Liu et al., 2010). In this situation, these employees may try to avoid proactive behavior in order to avoid risks. Based on the above analysis, job insecurity impacts the employees’ proactive behavior, which may be positive or negative. Therefore, the following hypothesis is proposed:

Hypothesis 1: Job insecurity significantly affects employees’ proactive behavior.

### The Mediating Role of Cognitive Appraisal

Many current researchers, such as De Witte et al. (2016), have proposed the idea of job insecurity-stress to explain further the way that job insecurity affects the relevant outcome variables, in which cognitive appraisal of stress plays a vital role. However, the cognitive appraisal of job insecurity in the relationship between job insecurity and proactive behavior is rarely studied. The cognitive appraisal of job insecurity and job insecurity are two completely different concepts. The cognitive appraisal can be defined as a personal appraisal of a particular situation (Paškvan et al., 2016), and the results of the appraisal will vary significantly according to individual characteristics. Employees’ cognitive appraisal of job insecurity can be either positive (challenge appraisal) or negative (hindrance appraisal) (Charkhabi, 2019).

Cognitive appraisal theory can provide theoretical support for the role of cognitive appraisal in the relationship between job insecurity and proactive behavior. Cognitive appraisal theory describes a subjective process involving cognitive appraisal and coping response (Lazarus and Folkman, 1987). According to the cognitive appraisal theory, stress exists in the combination of people and the environment, which stems from the cognitive evaluation of the environment (Broekens et al., 2008). Such cognitive appraisal leads people to think that there is an imbalance between the environment and the individual’s ability to cope with the environment. They believe that the environment is a burden on or beyond the scope of their ability and has an impact on them (Lazarus and Folkman, 1987). The appraisal process of the combination of human and environment involves two important processes: primary appraisal and secondary appraisal. Primary appraisal is the perception of the threat of an event or environment, a process of determining whether an event or environment is an obstacle or a challenge (Webster et al., 2011). If the event or environment is considered to be threatening, it will be evaluated as an obstacle. The secondary appraisal assesses an individual’s ability to cope with or deal with situations (Vander Elst et al., 2014), that is, to assess whether and to what extent they can take effective countermeasures.

Based on cognitive appraisal theory, the stress caused by job insecurity can be evaluated as a hindrance or challenge. Faced with the threat of losing the job or job characteristics, individuals will make a primary appraisal. When employees think that such job insecurity is not enough to pose a significant threat to their situation or even can help them grow and achieve future achievements, they tend to evaluate job insecurity as a challenge. This kind of challenge appraisal means that employees can weaken or even eliminate the threat that they may lose their job or job characteristics in the future through their efforts. These employees will work more actively and hard. While doing their work well, they will further internalize their roles, incorporate or define organizational citizenship behavior as job responsibilities within their roles, break the original job boundaries, take the initiative to assume responsibilities (Beck et al., 2014), give full play to their subjective initiative and show more proactive behavior. On the contrary, if employees believe that job insecurity causes tremendous pressure or damages potential interests and hinders the realization of goals, such situation will not only affect the physical and mental health of employees (Chirumbolo et al., 2017; Lastad et al., 2018), but also hinder their personal development, and employees will tend to make hindrance appraisal of job insecurity. Individuals who make a hindrance appraisal to the environment are more inclined to take a negative response. In this case, these employees not only do not show proactive behavior but are more passive. Therefore, the following hypotheses are proposed:

Hypothesis 2a: Challenge appraisal of job insecurity mediates the relationship between job insecurity and proactive behavior.
Hypothesis 2b: Hindrance appraisal of job insecurity mediates the relationship between job insecurity and proactive behavior.

### The Moderating Role of Self-Efficacy

Previous studies have shown that individual differences in the cognitive processes play an important role in the influence of stressors on related outcomes (Semmer, 2003). Self-efficacy refers to the degree of confidence that an individual can perform a certain behavior to produce a certain desired result.” That is, the degree of confidence an employee believes that he or she can weaken or even eliminate the threat of losing his or her job or job characteristics through taking initiative. This concept is consistent with the secondary appraisal process to some extent. At the same time, some studies have found that self-efficacy can ease the relationship between stress and related outcomes (Schaubroeck et al., 2000) and is an important psychological resource for coping with environmental pressure (Hobfoll et al., 1990).

Combined with social cognitive theory and cognitive appraisal theory, employees with higher self-efficacy have stronger self-confidence to cope with the pressure of job insecurity and believe that they can complete the tasks efficiently and effectively. These employees are more inclined to evaluate job insecurity as a hindrance. On the contrary, employees with lower self-efficacy are not confident enough about their workability, feel that they can’t complete tasks to meet specific requirements, and think that job insecurity brings blow and damage, and are more inclined to evaluate job insecurity as a hindrance. In conclusion, individuals with higher self-efficacy are more likely to make a challenge appraisal of job insecurity, while individuals with lower self-efficacy are more likely to make a hindrance appraisal of job insecurity. Therefore, the following hypotheses are proposed:

Hypothesis 3a: Self-efficacy moderates the relationship between job insecurity and challenge appraisal.
Hypothesis 3b: Self-efficacy moderates the relationship between job insecurity and hindrance appraisal.

Further, it is known from hypotheses 2a, 2b and hypotheses 3a, 3b that the relationship between job insecurity and proactive behavior is transmitted through cognitive appraisal. At the same time, self-efficacy may affect the relationship between employees’ job insecurity and proactive behavior via the cognitive appraisal, forming a moderated mediating model. Therefore, the moderated mediating effect hypotheses are proposed:

Hypothesis 4a: Self-efficacy moderates the mediating role of challenge appraisal in the relationship between job insecurity and proactive behavior.
Hypothesis 4b: Self-efficacy moderates the mediating role of hindrance appraisal in the relationship between job insecurity and proactive behavior.

**FIGURE 1:**
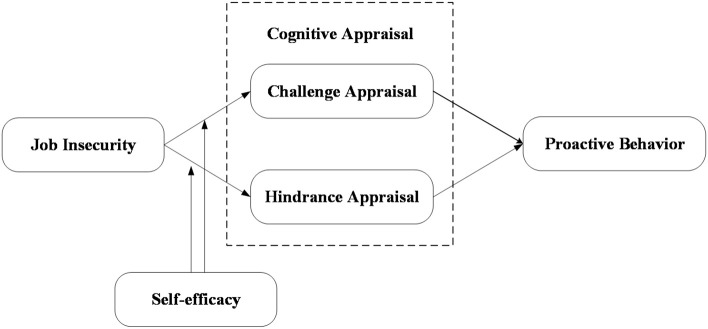
Research model.

Based on the above hypothesis, the theoretical model of the influence and mechanism of job insecurity on employees’ proactive behavior in this study is shown in Figure 1.

## Materials and Methods

### Sample and Procedures

The data of this study were obtained by questionnaire survey. The participants of the survey were employees of enterprises. The questionnaires were distributed online by a professional market research firm from August to September 2020, covering industries including finance, sales, construction, manufacturing, real estate, and administrative institutions. In order to improve the efficiency of the questionnaire, we promise that all the data collected will only be used for academic research and will not be disclosed. In the end, a total of 348 questionnaires were collected. We have set the survey objects as enterprise employees before the questionnaire was issued. After the invalid questionnaires were systematically excluded according to this standard, a total of 257 valid questionnaires were collected, with an effective rate of 73.85%. The sample structure is shown in Table 1.

**TABLE 1:** Sample structure (*n* = 257).

Variable	Features	Sample size	Ratio
Gender	Male	124	48.20%
	Female	133	51.80%
Age	Under the age of 25	55	21.40%
	25 to 35 years old	155	60.30%
	36 to 45 years old	45	17.50%
	More than 45 years old	2	0.80%
Educational background	High school degree and below	6	2.30%
	Junior college degree	29	11.30%
	Bachelor’s degree	200	77.80%
	Master’s degree or above	22	8.60%
Work experience	Less than 1 year	18	7.00%
	1 to 5 years	89	34.60%
	6 to 10 years	97	37.70%
	More than 10 years	53	20.06%

### Measures

All variables in this study were measured using the existing validated scale. In order to ensure that the scale items are more in line with the current research background, we modified some of the scale items and then invited five employees of the enterprise to try to fill in the questionnaire and modify the doubtful places to form the initial questionnaire of this study. All scales were scaled using a 5-point Likert scale, with a scale of 1 to 5 indicating “strongly disagree” to “strongly agree.”

### Job Insecurity

Using the scale adapted from Hellgren et al. (1999) to measure, including two dimensions: quantity job insecurity and quality job insecurity, a total of seven items. One of the quantity job insecurity items was, “I feel uneasy about losing my job in the future.” One of the quality job insecurity items was, “I think the organization will increase my job challenge in the future.” In this study, the reliability (Cronbach’s α) was 0.821.

### Self-Efficacy

The scale of general self-efficacy developed by Schwarzer et al. (1997) was adopted for measurement, consisting of ten items, such as “I can always solve difficult problems if I try my best” and “I am confident that I can effectively deal with anything unexpected.” The reliability (Cronbach’s α) was 0.872.

### Cognitive Appraisal

The cognitive appraisal of job insecurity scale which was further developed by Charkhabi (2018) was adopted for measurement, including two dimensions of the challenge and hindrance appraisal, with a total of six items. Challenge appraisal items such as “Job insecurity makes me focus on my job and perform better”; Hindrance appraisal items such as: “Job insecurity weakens my ability to focus on my job.” The reliability (Cronbach’s α) was 0.797 and 0.870.

### Proactive Behavior

The scale developed by Frese et al. (1997) was adopted for measurement, consisting of seven items, such as “I actively solve problems,” “I usually do more than I am required to do,” etc. The reliability (Cronbach’s α) was 0.838.

### Control Variables

Many previous studies have shown that individual characteristic variables are likely to have an impact on employees’ proactive behaviors (Parker et al., 2006). Therefore, demographic variables such as gender, age, educational background, and work experience are selected as control variables in this study.

## Results

### Common Method Deviation and Confirmatory Factor Analysis

Since all the questionnaire data in this study were obtained by the same subject in the way of self-evaluation, there may be a problem of common method deviation. Harman single factor test was used to test the possible common method bias. The results showed that seven factors were eutectoid from the unrotated principal component analysis. The variance interpretation rate of the first factor was 32.583%, less than 40%. Therefore, it could be considered that there was no serious common method deviation.

Before hypothesis testing, confirmatory factor analysis (CFA) of the data is needed. In this study, MPLUS 7.4 software was used for CFA to test the validity of the questionnaire. The test results of construction reliability and aggregation validity are shown in Table 2. Firstly, the construction reliability (CR) of each variable is greater than 0.8, indicating that the CR of the questionnaire in this study meets the requirements. Secondly, the average variance extraction (AVE) of each variable is greater than 0.5, indicating that the questionnaire had good aggregation validity. Thirdly, it can be seen from Table 3 that the direct correlation coefficient of all variables is less than the root mean square of their AVE, which reflects that the discriminant validity of each scale meets the requirements.

**TABLE 2:** Construction reliability and aggregation validity.

Variable	CR	AVE
Job insecurity	0.938	0.686
Self-efficacy	0.949	0.652
Challenge appraisal	0.891	0.733
Hindrance appraisal	0.898	0.746
Proactive behavior	0.937	0.679

**TABLE 3:** Mean, standard deviation, and correlation coefficient of variables.

Variable	Mean	*SD*	1	2	3	4	5	6	7	8	9
1. Gender	1.52	0.501									
2. Age	1.98	0.649	–0.059								
3. Educational background	2.93	0.536	–0.046	–0.016							
4. Work experience	2.72	0.870	–0.060	0.749^**^	–0.053						
5. Job insecurity	2.34	0.707	0.019	–0.092	–0.108	–0.120	(0.837)				
6. Self-efficacy	3.62	0.638	–0.090	0.111	0.082	0.117	−0.550^**^	(0.808)			
7. Challenge appraisal	3.37	0.880	–0.089	–0.058	–0.027	–0.004	−0.210^**^	0.440^**^	(0.856)		
8. Hindrance appraisal	2.61	1.014	–0.047	−0.168^**^	–0.045	−0.164^**^	0.324^**^	−0.302^**^	−0.382^**^	(0.864)	
9. Proactive behavior	3.81	0.629	–0.105	0.175^**^	0.080	0.209^**^	−0.499^**^	0.741^**^	0.487^**^	−0.290^**^	(0.819)

^1^n = 257, *p < 0.05, **p < 0.01, and ***p < 0.001. The values in brackets are AVE root mean square of each scale.

### Descriptive Statistics Analysis

The mean, standard deviation, and correlation coefficients of the variables are shown in Table 3. It can be seen from the table that there was a significant negative correlation between job insecurity and employees’ proactive behavior (*r* = −0.499, *p* < 0.05). Job insecurity was significantly negatively correlated with challenge appraisal (*r* = −0.210, *p* < 0.05) and was significantly positively correlated with hindrance appraisal (*r* = 0.324, *p* < 0.05). The self-efficacy was positively correlated with employees’ challenge appraisal of job insecurity (*r* = 0.440, *p* < 0.05) and was negatively correlated with employees’ hindrance appraisal of job insecurity (*r* = −0.302, *p* < 0.05). Employees’ challenge appraisal (*r* = 0.487, *p* < 0.05) and hindrance (*r* = −0.290, *p* < 0.05) appraisal of job insecurity were significantly correlated with proactive behavior. This is consistent with the expectation of this study, and Hypothesis1 is preliminarily verified, which also lays a foundation for subsequent hypothesis testing.

Finally, in order to test the structural validity of the questionnaire, the single-factor model, two-factor model, three-factor model, four-factor model, and five-factor model were tested, respectively, as shown in Table 4. According to the results, the fitting effect of the five-factor model is better and obviously better than other models, which reflects the good structural validity of the questionnaire.

**TABLE 4:** Structural validity.

Model	χ^2^	df	χ^2^/df	RMSEA	CFI	TLI
Single-factor model	2049.821	436	4.701	0.120	0.538	0.507
Two-factor model^a^	984.325	433	2.273	0.109	0.668	0.639
Three-factor model^b^	977.323	431	2.268	0.080	0.844	0.831
Four-factor model^c^	866.324	429	2.019	0.075	0.869	0.858
Five-factor model	848.215	428	1.982	0.064	0.874	0.863

*^a^Combining self-efficacy, challenge appraisal, hindrance appraisal, and proactive behavior into one factor.*

*^b^Combining job insecurity, challenge appraisal, and hindrance appraisal into one factor.*

*^c^Combining challenge appraisal and hindrance appraisal into one factor.*

### Hypothesis Test

Based on SPSS 21.0, this study used hierarchical regression analysis to test the mediating effect of cognitive appraisal of job insecurity and the moderating effect of self-efficacy. The test results are shown in Table 5.

**TABLE 5:** Hierarchical regression analysis.

Variable	Challenge appraisal			Hindrance appraisal			Proactive behavior			
	Model 1	Model 2	Model 3	Model 4	Model 5	Model 6	Model 7	Model 8	Model 9	Model 10
Gender	–0.091	–0.056	–0.041	–0.063	–0.076	–0.047	–0.088	–0.085	–0.048	–0.093
Age	–0.126	–0.148	–0.141	–0.102	–0.094	–0.080	0.035	0.035	0.087	0.022
Educational background	–0.054	–0.064	–0.068	–0.020	–0.016	–0.024	0.086	0.031	0.053	0.029
Work experience	0.055	0.049	0.048	–0.056	–0.054	–0.056	0.182	0.122	0.100	0.115
Job insecurity	−0.219^***^	0.035	0.036	0.307^***^	0.213^**^	0.215^**^		−0.476^***^	−0.386^***^	−0.436^***^
Self-efficacy		0.471^***^	0.447^**^		−0.174^*^	−0.218^**^				
Challenge appraisal									0.409^***^	
Hindrance appraisal										−0.129^*^
Job insecurity × Self-efficacy			0.103			0.196^**^				
*R* ^2^	0.044	0.196	0.203	0.112	0.130	0.162	0.045	0.266	0.424	0.278
*F*	3.352^**^	11.400^***^	10.297^***^	7.467^***^	7.368^***^	8.089^***^	4.039^**^	19.576^***^	32.357^***^	17.443^***^

^a^n = 257, *p < 0.05, **p < 0.01, and ***p < 0.001. Data in the table are normalized regression coefficients.

For the test of mediating effect, according to the suggestion of Baron and Kenny (1986), a three-step regression method was adopted. First of all, the control variables and independent variables regression were carried out for the mediated variable of challenge appraisal and hindrance appraisal, respectively. According to Model 1 and Model 4, job insecurity has a significant effect on employees’ challenge appraisal (*r* = −0.219, *p* < 0.001) and hindrance appraisal (*r* = 0.307, *p* < 0.001). Secondly, through the regression of control variables and the independent variable on the outcome variable, Model 8 shows that job insecurity has a significant negative effect on employees’ proactive behavior (*r* = −0.476, *p* < 0.001), and hypothesis 1 was verified. Finally, the mediating variable challenge appraisal and hindrance appraisal were added into the regression equation, respectively. The results showed that, in Model 9 and Model 10, the effect of job insecurity on proactive behavior was weakened, and the mediating variable challenge appraisal (*r* = 0.409, *p* < 0.001) and hindrance appraisal (*r* = −0.129, *p* < 0.05) had a significant effect on proactive behavior, indicating that employees’ cognitive appraisal of job insecurity played a partial mediating role between job insecurity and proactive behavior. Hypothesis 2a and Hypothesis 2b were also verified.

**FIGURE 2:**
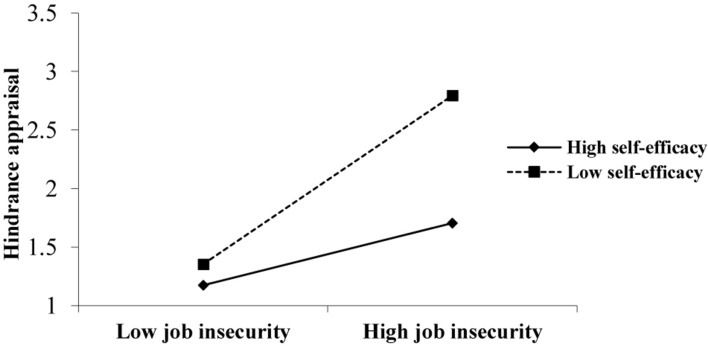
The moderating effect of self-efficacy on the relationship between job insecurity and hindrance appraisal of job insecurity.

In order to test the moderating effect of self-efficacy, according to the suggestion of Edwards and Lambert (2007), the challenge and hindrance appraisal of job insecurity were, respectively, taken as the outcome variables. The job insecurity, self-efficacy, and the interaction between job insecurity and self-efficacy were successively added into the regression equation in turn. Model 3 shows that the interaction between job insecurity and self-efficacy has no significant effect on challenge appraisal, hypothesis 3a has not been verified. According to Model 6, the interaction between job insecurity and self-efficacy had a significant positive effect on hindrance appraisal (*r* = 0.196, *p* < 0.01), hypothesis 3b has been verified. Further, we drew a schematic diagram of the moderating effect of self-efficacy on the relationship between job insecurity and employees’ hindrance appraisal, as shown in Figure 2. It can be seen from the figure that self-efficacy has a negative moderating effect on the positive relationship between job insecurity and hindrance appraisal of job insecurity. That is to say, the lower the level of self-efficacy, the more likely the employees to make a hindrance appraisal of job insecurity.

For the test of moderated mediating effect, we used the approach of Preacher et al. (2007) to test the moderating effect of self-efficacy on the mediating effect of cognitive appraisal between job insecurity and proactive behavior through the Process program of SPSS software. As indicated in Table 6, job insecurity had a stronger mediating effect on proactive behavior via hindrance appraisal when the self-efficacy had low levels (Effect = 0.571, SE = 0.121, CI = 0.333–0.809) rather than high levels (Effect = 0.325, SE = 0.098, CI = 0.131–0.519). H4b was verified.

**TABLE 6:** The test of moderated mediating effect (hindrance appraisal).

Moderator	Level	Effect	Boot SE	Boot *p*	CI
Self-efficacy	High (+1 SD)	0.325	0.098	0.000	0.131–0.519
	Low (−1 SD)	0.571	0.121	0.000	0.333–0.809

*n = 257, N = 5000, CI: Confidence Interval, CI = 95%.*

## Discussion

Based on social cognitive theory and cognitive appraisal theory, this study tests the effects of job insecurity on proactive behavior and its mechanism. Through the analysis of 257 valid questionnaires, the following research findings can be derived:

First, job insecurity has significant negative effects on employees’ proactive behavior. Although Teng et al. (2019) found that job insecurity can promotes employees’ innovative behavior. This finding is consistent with the conclusions of most of those previous researches on the impact of job insecurity on positive behaviors, that is, job insecurity has a significant negative impact on employees’ positive behaviors, such as voice behavior (Yin and Chung, 2019) and extra-role behavior (Yu et al., 2021). It may be related to the increase of psychological stress and the decrease of organizational commitment of employees (Chirumbolo et al., 2017) caused by job insecurity.

Second, the cognitive appraisal of job insecurity plays a partial mediating role between job insecurity and proactive behavior. In particular, job insecurity negatively impacts employees’ challenge appraisal, and the challenge appraisal of job insecurity can promote employees’ proactive behavior. Obviously, challenge appraisal of job insecurity moderates the negative effect of job insecurity on employees’ proactive behavior. At the same time, job insecurity positively impacts employees’ hindrance appraisal, and the hindrance appraisal of job insecurity can inhibit employees’ proactive behavior. Obviously, hindrance appraisal of job insecurity also moderates the negative effect of job insecurity on employees’ proactive behavior. This finding supports previous research that cognitive appraisal plays a mediating role between job insecurity and related outcomes (Charkhabi, 2019). This is because the cognitive appraisal of employees’ job insecurity will inevitably affect their physical and mental health, work attitude, and behavior (Gomes et al., 2016; Charkhabi, 2019). It also confirmed the model in which cognitive appraisal partially mediating stressor-outcome relationship (Webster et al., 2011).

Third, self-efficacy played a moderating role in the relationship between job insecurity and hindrance appraisal of job insecurity. The higher the self-efficacy, the more significant the moderating effect was. This further validates the content of the cognitive appraisal theory and is consistent with the findings of Schaubroeck et al. (2000) that self-efficacy can alleviate the relationship between stress (job insecurity) and related outcomes (cognitive appraisal).

Fourth, self-efficacy can moderate the mediating effect of cognitive appraisal of job insecurity on the relationship between job insecurity and proactive behavior. The higher the self-efficacy, the stronger the mediating effect of hindrance appraisal of job insecurity and proactive behavior.

## Conclusion and Implications

This study explores the influence of job insecurity on employees’ proactive behavior and explains the phenomenon that different employees in organizations have different responses to job insecurity. It fills the gap in the academic research on the relationship between job insecurity and proactive behavior. To test our hypothesis, 257 valid questionnaires were analyzed using the hierarchical regression analysis and the Bootstrap method. Most of the hypotheses have been verified. The results show that job insecurity negatively impacts employees’ proactive behavior. Cognitive appraisal of job insecurity plays a mediating role between job insecurity and proactive behavior. Self-efficacy not only moderated the relationship between job insecurity and hindrance appraisal, but also moderated the mediating role of hindrance appraisal between job insecurity and proactive behavior.

### Theoretical Implications

Based on the perspectives of social cognition theory and cognitive appraisal theory, we associated job insecurity with employees’ proactive behavior, which expands the perspective and boundary of the research on job insecurity and proactive behavior. Previous studies on job insecurity are mostly based on social exchange theory and social identity theory to discuss the impact of job insecurity on employees’ internal psychological intentions, such as well-being (De Witte et al., 2016) and organizational commitment (Chirumbolo et al., 2017), while fewer researchers paid attention to the impact of job insecurity on employees’ behavioral intentions and work behaviors. In recent years, with the attention of all walks of life to innovation, some researchers have discussed the relationship between job insecurity and employees’ innovative behavior and creativity (Teng et al., 2019). However, in today’s increasingly fierce market competition, organizations urgently need employees to exert their subjective initiative to cope with the torrent of market competition. Proactive behavior has become one of the key factors that determine the success or failure of organizations today. This study takes the proactive behavior as a result variable of job insecurity, from the perspective of employee’s cognitive appraisal of job insecurity, discusses the influences of job insecurity on employees’ proactive behavior, which provides a new theoretical perspective for the study of behavior and enriches the previous research on the result variables of job insecurity.

At the same time, this study combined individual factors with environmental factors, explores the mechanism of job insecurity on proactive behavior, and explained the “black box” of job insecurity and employees’ proactive behavior association. As a common source of work stress in organizations (Richter et al., 2014), job insecurity will inevitably impact employees’ work behavior. However, the mechanism of its impact on employees’ proactive behavior still needs to be further studied. So, this study introduced employees’ cognitive appraisal of job insecurity as a mediating variable, took individual factor self-efficacy as a moderating variable. Combining environmental factors with individual factors, this study discusses the effect of job insecurity on proactive behavior. Job insecurity has a direct effect on proactive behavior and affects employees’ proactive behavior through their cognitive appraisal of job insecurity, in which self-efficacy plays a moderating role.

### Practical Implications

By constructing a moderated mediation model, this paper explores the impact of job insecurity on employees’ proactive behavior. It explains the phenomenon that different employees in an organization have different performances in the face of job insecurity. In today’s organizations, different employees react differently when facing the same pressure. Some employees are lazier and slack off in the face of job insecurity, while others are relatively optimistic. This study conclusion explains the reason for this organizational phenomenon. In the face of job insecurity, employees make cognitive appraisals to evaluate this pressure as a challenge or hindrance. When job insecurity harms proactive behavior, challenge appraisal of job insecurity can alleviate the negative impact. Employees will still properly play the subjective initiative. While hindrance appraisal of job insecurity will further deepen this negative impact, and employees will correspondingly less proactive.

It provides new ideas for the organization to stimulate the employees to show more proactive behavior. Today, job insecurity has widely existed in all kinds of enterprises, and no enterprise can eliminate the job insecurity. Job insecurity hurts employees, to a certain extent, can promote the innovation behavior of employees. In this case, only as much as possible to weaken the negative impact of job insecurity on proactive behavior. The conclusion of this study shows that to stimulate the subjective initiative of employees to the maximum extent: On the one hand, it is necessary to pay attention to the self-confidence and self-efficacy of employees themselves from the perspective of individual employees. Enterprises can carry out a variety of targeted training according to the different situations of employees to enhance their working ability and thus enhance their sense of self-efficacy. Simultaneously, contingency management should be adopted to give appropriate recognition to employees’ work and cultivate their work confidence. On the other hand, from the perspective of the organizational environment, employees’ job insecurity should be appropriately reduced to reduce the impact on employees’ work enthusiasm. Enterprises can reduce employees’ job insecurity by providing more organizational support for employees and creating an organizational atmosphere in which leaders care about employees.

### Limitations and Future Research

This study has made some contributions in theory and management practice, but there are still the following research limitations:

First of all, the sample data of this study was collected from multiple industries enterprise employees, is not restricted to a particular industry. However, industry employees’ job insecurity and self-efficacy will vary by industry, especially the different industries affected by the outbreak of the COVID – 19 degree has the various difference. So, it is significant to study the job insecurity of employees for a specific industry (such as manufacturing industry, foreign trade industry, etc.). Future research can limit specific industries to carry out relevant research so that enterprises in different industries can formulate more targeted management plans according to their actual situation.

Secondly, the data of this study come from employees’ self-evaluation at the same point in time, although the data analysis has passed the Harman single factor test proves that there is no serious problem of common method deviation, it is still a cross-section data. Even if most of the research hypotheses of this study were supported, also cannot truly reflect the causal relationship between variables. In the future, we can try to collect longitudinal tracking data for research to further effectively verify the mechanism of job insecurity on employees’ proactive behavior.

Finally, the theoretical model of this study is only from the individual level employees, discusses the effects and mechanism of job insecurity on proactive behavior, does not take into account the organization level and other factors that influence the employee’s work behavior. However, employees’ work behavior is influenced by many factors. Future research can focus on the effects and its mechanism of organizational-level factors (such as the organizational atmosphere) and the leadership level factors (such as leadership style) on proactive behavior, and further expand the research on influencing factors and its mechanism of employees’ proactive behavior.

## Data Availability Statement

The original contributions presented in the study are included in the article/supplementary material, further inquiries can be directed to the corresponding author.

## Ethics Statement

Written informed consent for participation was not required for this study in accordance with the national legislation and the institutional requirements. Written informed consent was implied via completion of the survey.

## Author Contributions

XY developed the research model. ML collected the data, analyzed the data, and co-drafted the manuscript. HZ co-drafted the manuscript and revised it critically for important intellectual content. All authors contributed to the article and approved the submitted version.

## Conflict of Interest

The authors declare that the research was conducted in the absence of any commercial or financial relationships that could be construed as a potential conflict of interest.

## Publisher’s Note

All claims expressed in this article are solely those of the authors and do not necessarily represent those of their affiliated organizations, or those of the publisher, the editors and the reviewers. Any product that may be evaluated in this article, or claim that may be made by its manufacturer, is not guaranteed or endorsed by the publisher.
